# Placenta Prævia; Its History and Treatment

**Published:** 1861-12

**Authors:** 


PLACENTA PRÆVIA; ITS HISTORY AND TREATMENT. By WILLIAN READ, M. D., Member of the Massachusetts Medical Society.

However justifiable the complaint of want of interest and cultivation in the branch of medical service to which this volume belongs, may have been in time past, the evidence of its growing importance and rapid advancement in the last quarter of a century, is proof of the industrious interest taken in it by a large body of the best members of our profession, at the present. Indeed, no branch is cultivated with more zeal than obstetrics, and it is gratifying, and we think, truthful, to say, that none has advanced with more rapid strides, in the last quarter of a century. Many illustrious names, and almost innumerable monographs, as well as general treatises, could be mentioned in proof of this statement, but it is not necessary to do so now.

Since the lucid and masterly efforts of Rigby, the elder, to clear up the hitherto obscure subject of uterine hæmorrhage, it has undergone the most scrutinizing investigations at the hands of many very competent men, and strange to say, notwithstanding the various deviations, which, from time to time, have been made from the practical doctrine, taught by him, we are now almost exactly where he left the subject in point of fact. The volume of Dr. READ is creditable to his industry, and proves the great interest he has manifested for his subject, and the vast knowledge he possesses of its literature and nature, and will well pay for its perusal. We think there can be but one opinion of the main practical teachings of it, and no question of the plausibility of the theories discussed. The former are excellent and reliable, and the latter maintained with as many facts and as much logic, as are necessary to convince the most of his readers. The following are the particular conclusions at which he has arrived, viz:

1. The danger to the mother in placenta prævia increases the nearer the approach to full term of pregnancy. It is better, therefore, to terminate labor when it begins prematurely, than to endeavor to conduct the pregnancy to full term.

2. The danger to the mother is less when the os uteri is completely covered, than when a portion only is involved with the attachment of the placenta, and least of all when the attachment is completely central.

3. The *condition* of the mother is of more importance than the absolute quantity of blood lost. A small amount will affect some persons more than a larger quantity will others. The first evident approach of danger, as syncope or its premonitos should serve as a signal for action,—the chances of recovery becoming momentarily less, when symptoms of failure in the system have commenced.

4. When the pains are vigorous, and likely to be so, rupturing the membranes may be sufficient in most instances to check the hæmorrhage. When the pains are not efficient, he would oppose this measure.

5. The danger to the mother is materially increased by artificial delivery, not so much on account of the operation itself, as the exhausted condition of the mother at the time it is usually done.

6. It should therefore be the aim of the practitioner, to deliver before great exhaustion has occurred.

7. If, from the progress of the case, or the conditions of labor, a resort to artificial labor must finally be had, it should not be delayed an instant beyond the time when the dilatation or dilatability of the os uteri permits the introduction of the hand into the uterus.

8. When, from the rapidly failing condition of the mother, or the presence of any cause artificial delivery is rendered impossible, the placenta should be wholly separated from the uterus, and means used to restore her, until delivery is practicable.

9. The tampon may be used to check flowing before the os uteri is dilated or dilatable, care being taken to not allow it to delay the delivery.

10. Ergot should not be used when an operation is likely to become necessary, unless we can wait long enough for its effects to pass off, or at a time when its effects will not be established, until after the operation is over.

11. In cases where the exhaustion is excessive, and version is the only alternative, after the feet have been brought down, the body should be left undelivered until the uterus has been roused to contractions, or at least, withdrawn slowly.

The volume is published by order of the Massachusetts Medical Society, for the benefit of its Fellows, by Lippincott & Co., Philadelphia, and is a handsome, well-printed book of some 340 pp.

